# Locally advanced thymoma; does neoadjuvant chemotherapy make a difference?

**DOI:** 10.1186/s13019-023-02357-4

**Published:** 2023-08-18

**Authors:** Riad Abdel Jalil, Farah A. Abdallah, Zeinab Obeid, Ahmad Khaled Harb, Mohamad K. Abou Chaar, Tariq Bassem Shannies, Ahed El-Edwan, Hussam Haddad, Azza Ghraibeh, Ahmad Abu-Shanab

**Affiliations:** 1 Department of Thoracic Oncology, King Hussein Cancer Center, Queen Rania Al Abdullah Street, P.O. Box 1269, Amman, 11941 Jordan; 2 Department of Research, King Hussein Cancer Center, Queen Rania Al Abdullah Street, P.O. Box 1269, Amman, 11941 Jordan; 3 Department of Surgery, King Hussein Cancer Center, Queen Rania Al Abdullah Street, P.O. Box 1269, Amman, 11941 Jordan; 4 Department of Anesthesia, King Hussein Cancer Center, Queen Rania Al Abdullah Street, P.O. Box 1269, Amman, 11941 Jordan; 5 Department of Pathology, King Hussein Cancer Center, Queen Rania Al Abdullah Street, P.O. Box 1269, Amman, 11941 Jordan; 6 Department of Radiology, King Hussein Cancer Center, Queen Rania Al Abdullah Street, P.O. Box 1269, Amman, 11941 Jordan

**Keywords:** Thymoma, Neoadjuvant chemotherapy, Radiographic effect, Histopathological effect

## Abstract

**Background:**

Regardless of its rare occurrence, Thymoma remains the most frequently encountered primary tumor of the anterior mediastinum comprising about 50% of all masses in the region. Surgical resection, via thymectomy, remains the mainstay treatment modality. In locally advanced and borderline resectable tumors, neoadjuvant chemotherapy (NACT) may be utilized to increase the chance of R0 resection, raising the question of its efficacy and safety.

**Methods:**

Demographic and clinical data from patients who presented to a tertiary cancer center between January 2015–October 2021 with a diagnosis of thymoma and underwent curative surgical resection was collected. Computed tomography scan was used to delineate clinical staging, tumor size and to detect post-therapeutic variations in tumor burden. The response evaluation criteria in solid tumors (RECIST) was used to classify the effect of NACT on tumor burden. The pathological response was determined by measuring the percentage of necrotic tissue.

**Results:**

A total of 23 patients were diagnosed with thymoma. Most patients were male with a mean age 46 (± 15) years at diagnosis. The most common clinical stage was stage II with 5 patients (22%). A total of 12 patients had NACT as compared to 11 patients who had upfront surgery. The mean change in tumor volume was 165 cm^3^ (p = 0.079) and the change in and maximum diameter was 1.53 ± 1.49 cm (p < 0.01). The effect of NACT on tumor burden based on RECIST criteria was minimal as 8 patients had stable disease. Based on pathological findings, the average necrotic portion of the tumor was 39.5% (p = 0.152). The overall survival rate is 95.65%, mean survival was 115 months (4–125). Recurrence occurred in 5 patients. The NACT group had a higher risk for recurrence (4; 33.3%) with a mean survival of 43.8 months compared to 59.6 months in those who did not receive induction therapy.

**Conclusions:**

The exact role of induction chemotherapy in locally advanced thymoma patients remains controversial. NACT effect after utilizing radiological and pathological assessment tools was not found to significantly improve oncological outcomes compared to upfront surgery in locally advanced disease, with minimal radiologic and pathologic effect. To further demonstrate the impact of induction chemotherapy, we recommend multicentric collaborative studies.

## Introduction

Tumors of the thymus gland, termed thymomas, are rare but remain the most frequently encountered primary tumor of the anterior mediastinum comprising about 75–80% of all masses in the region [1]. This constitutes Thymic epithelial tumors (TETs), originating from the epithelial lining of the thymus [2]. According to World Health Organization Classification, thymoma has 8 major subtypes (type A, atypical type A variant, Type AB, Type B1. Type B2, Type B3, micronodular thymoma with lymphoid stroma, and metaplastic thymoma) with the addition of three rare subtypes (microscopic thymoma; sclerosing thymoma, lipofibroadenoma) [3], while Masoka –Koga staging is based on tumor invasiveness [4] Surgical resection, via thymectomy, remains the mainstay treatment modality for early and recurrent cases [5]. In contrast, a multi-disciplinary approach of chemo-radiotherapy, followed by surgery is utilized in locally advanced or metastatic thymoma [6].

In locally advanced and borderline resectable tumors, Masaoka-Koga stage III/IVA, induction chemotherapy may be warranted to increase the rate of complete resection with negative margins [7].

Moreover, evidence on the outcomes of individuals with advanced illness who received induction treatment is inconsistent. Herein, we present comparison between two groups of patients who were diagnosed with thymoma and either underwent neoadjuvant therapy before surgical resection or had upfront surgery.

## Methods

Relevant data from patients who were diagnosed to have thymoma and treated at King Hussein Cancer Center between January 2015–October 2021 was accessed after obtaining institutional review board (IRB#). Electronic medical records were utilized to obtain ​​patients’ demographic data and clinical characteristics including; age, gender, smoking status, staging, treatment regimens, response, post-treatment, or post-operative events, and comorbidities. Immunological studies have been carried out to confirm co-existing Myasthenia Gravis (MG).

### Radiological evaluation

**Fig. 1:**
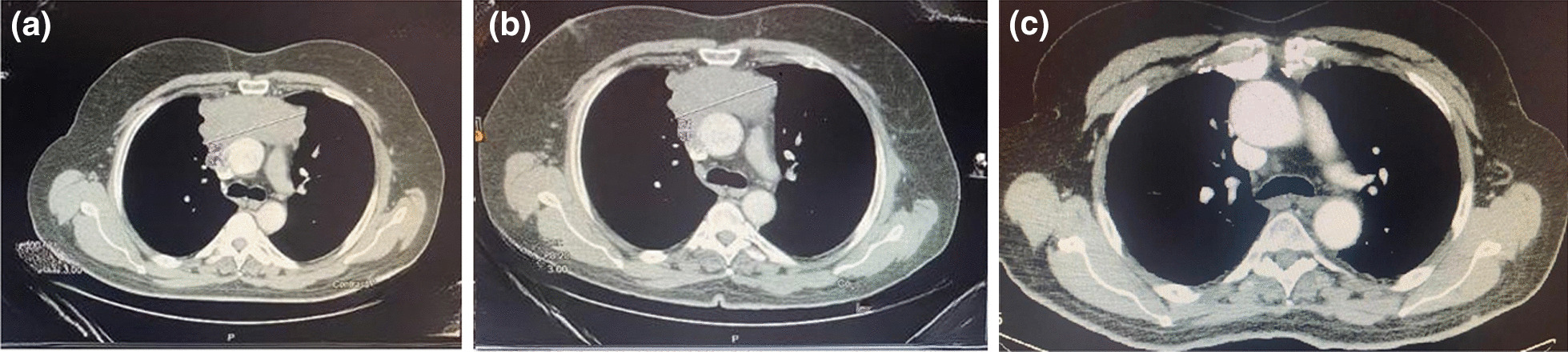
**a** Computed tomography (CT) of thymoma tumor pre-neoadjuvant chemotherapy. **b** Computed tomography (CT) of thymoma tumor post-neoadjuvant chemotherapy.  **c** Chest computed tomography (CT) after surgery

Contrast-enhanced computed tomography (CT) scan was used to delineate clinical staging, tumor size and to detect post-neoadjuvant chemotherapy treatment effect on tumor burden. The response evaluation criteria in solid tumors (RECIST) was used to classify the effect of neoadjuvant chemotherapy (NACT) on tumor burden as complete response (CR), partial response (PR), stable disease (SD) or progressive disease (PD). Comparison was made between pre-neoadjuvant therapy initiation and post-operatively. All images were evaluated by a single expert radiologist (Fig. 1).

### Pathological evaluation

**Fig. 2:**
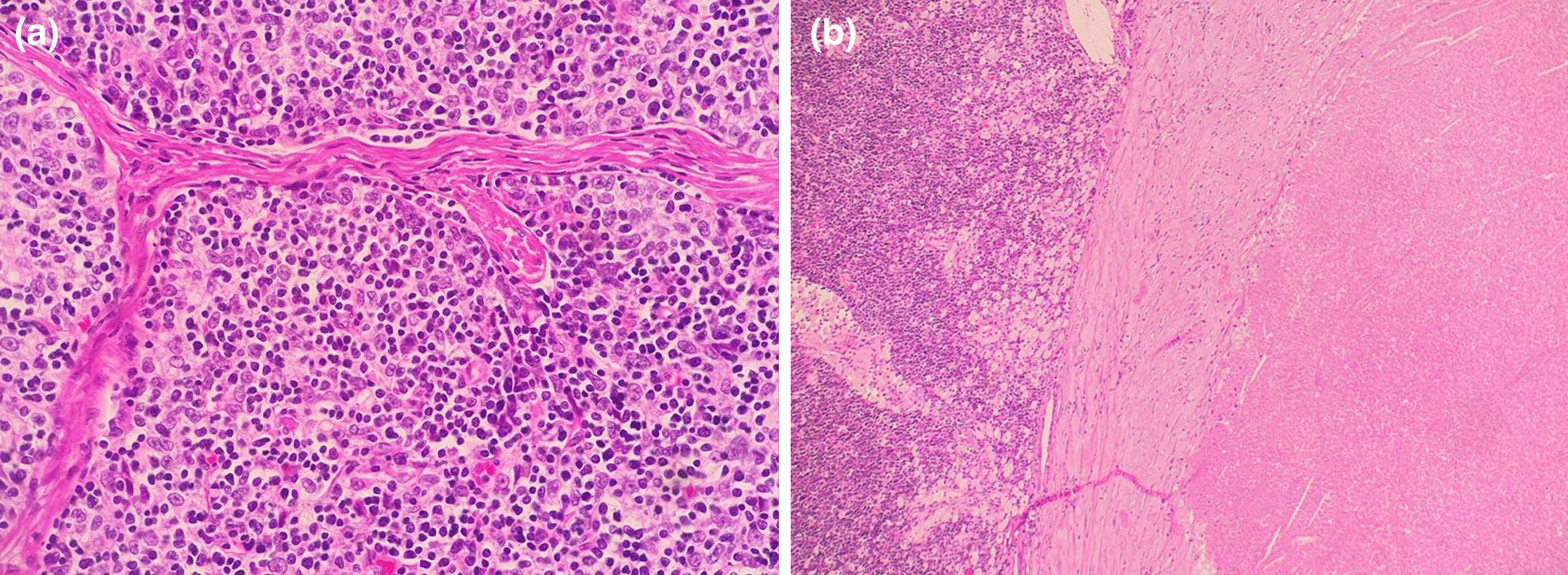
**a** Histological view of thymoma tissue. **b** Necrotic thymoma histology

All histological specimens were received immediately postoperatively in formalin providing information about histological type, margins, regional lymph nodes, and the presence of lymphovascular invasion. The pathological response was determined by measuring the percentage of necrotic tissue (Fig. 2).

### Surgical approach

All patients underwent surgical resection of the thymoma either via open approaches utilizing the median sternotomy or thoracotomy. Some patients underwent minimally invasive with multiple or single port Video assisted thoracoscopic surgery (VATS).

## Results

A total number of 23 patients were included in this retrospective analysis.

The majority of the patients were male with a mean age of 46 ± 15 at diagnosis. Clinical stage distribution was found to be: stage I (3; 13%), stage II (5; 22%), stage III (3; 13%), stage IV (12; 52%). Myasthenia Gravis was diagnosed in 22% of patients.

Patients who received NACT were distributed among stage I (1, 8%), stage III (2, 16%) and stage IVA (9, 39%). Chemotherapy regimen was a combination of doxorubicin, cisplatin, and cyclophosphamide. Eleven patients underwent VATS tumor resection with two in the NACT group (18%). Negative resection margins were found in 18 (78%) patients (Table 1). A single patient received definitive radiation and 12 cycles of chemotherapy outside our center, developed chemotherapy-induced heart failure, and died immediately postoperatively. No other patients reported severe toxicity. It was noted that those who had advanced clinical stage (Stage IV) were more likely to receive neoadjuvant therapy (p = 0.013) and those who had an open thymectomy procedure were more likely to have neoadjuvant therapy (p = 0.002) as shown on Table 2.

**Table 1:** Patient characteristics

Variable	Number of patients n = 23
Age (years)	46.28 (± 15.3)
Gender	
Male	18 (78%)
Female	5 (22%)
Comorbidities^α^	
Diabetes mellitus	3 (13%)
Hypertension	3 (13%)
Other^β^	5 (22%)
Histologic subtypes	
A	1 (4%)
AB	4 (17%)
BI	1 (4%)
BII	8 (35%)
BIII	9 (39%)
Staging of Masaoka	
Stage I	3 (13%)
Stage II	5 (22%)
Stage III	3 (13%)
Stage IV	12 (52%)
Myasthenia graves	5 (22%)
Neoadjuvant chemotherapy	
Stage I	1 (8%)
Stage III	2 (16%)
Stage IIV	9 (75%)
Surgical approach	
VATS^Ω^	11 (48%)
Open	12 (52%)
Negative resection margin (R0)	18 (78%)

α Two patients had diabetes mellitus and hypertension simultaneously

β Included: hyperlipidemia, carcinoid syndrome, heart failure, atrial fibrillation, Immune thrombocytopenic purpura, and epilepsy

Ω Video-assisted thoracoscopic surgery

**Table 2:** Univariate analysis between those who received neoadjuvant therapy and those who did not

Variable	Total N = 23	Neoadjuvant		p Value
		Yes N = 12	No N = 11	
Gender				
Female	5	3 (60%)	2 (40%)	0.99
Male	18	9 (50%)	9 (50%)	
Negative resection margin				
No	5	4 (80%)	1 (20%)	0.317
Yes	18	8 (44%)	10 (56%)	
Smoking				
Missing	1	0	1	0.99
Ex-smoker	4	2 (50%)	2 (50%)	
No	12	7 (58%)	5 (42%)	
Yes	6	3 (50%)	3 (50%)	
Stage				
I	3	1 (33%)	2 (67%)	0.013
II	5	0	5 (100%)	
III	3	2 (67%)	1 (33%)	
IV	12	9 (75%)	3 (25%)	
Histology				
A	1	0	1 (9.1%)	0.99
AB	4	2(16.7%)	2 (18.2%)	
BI	1	0	1 (100%)	
BII	8	4 (50%)	4 (50%)	
BIII	9	6 (67%)	3 (33%)	
Surgical approach				
Open	12	10 (83%)	2 (17%)	0.002
VATS	11	2 (18%)	9 (82%)	
Adjuvant therapy				
No	8	5 (63%)	3 (38%)	0.667
Yes	15	7 (47%)	8 (53%)	
Complications				
No	17	8 (47%)	9 (53%)	0.640
Yes	6	4 (67%)	2 (33%)	
Lymphovascular invasion				
Missing	7	4 (57%)	3 (43%)	0.282
No	11	4 (36%)	7 (64%)	
Yes	5	4 (80%)	1 (20%)	
Myasthenia gravis diagnosis				
No	18	11 (61%)	7 (39%)	0.155
Yes	5	1 (20%)	4 (80%)	
Current status				
Alive	22	11 (50%)	11 (50%)	0.99
Dead	1	1 (100%)	0	
Recurrence				
No	18	8 (44%)	10 (56%)	0.317
Yes	5	4 (80%)	1 (20%)	
Cystic component				
Missing	3	2 (67%)	1 (33%)	0.474
No	18	10 (56%)	8 (44%)	
Yes	2	0	2 (100%)	

### Radiological evaluation

The mean change in tumor volume and maximum diameter was 165 cm^3^ (p = 0.079) and 1.53 ± 1.49 cm (p < 0.01) respectively (Table 3). Tumor diameter stratified according to stage showed a variation of 2.0 ± 1.6 cm in stage IVa (p = 0.02) and 1 ± 1.35 cm in stage III (p = 0.08), respectively. The effect of NACT on tumor burden based on RECIST criteria was minimal as 80% (n = 8) of patients had SD and the remaining 2 patients had PR and PD.

**Table 3:** Radiographic effect of NACT on thymic tumors

Variable	Measurement	p-Value
Change in tumor volume (mean)	165 cm^3^	0.079
Change in maximum diameter	1.53 cm ± 1.49	< 0.01
Tumor diameter		
Stage III	1 cm ± 1.35	0.08
Stage IV	2.0 cm ± 1.6	0.02

### Pathological evaluation

Based on pathological findings, the average necrotic portion of the tumor was 39.5%. Although the portion of necrosis was evidently higher in stage IVA (56% vs 23%), these values were not statistically significant (p = 0.152) as shown in Table 4.

**Table 4:** Histopathological effect of NACT on thymic tumors

NACT effect on tumors	No. of patients N = 12
Stable disease	8 (66%)
Partial response	1 (8%)
Progressive disease	1 (8%)

### Survival analysis

**Fig. 3:**
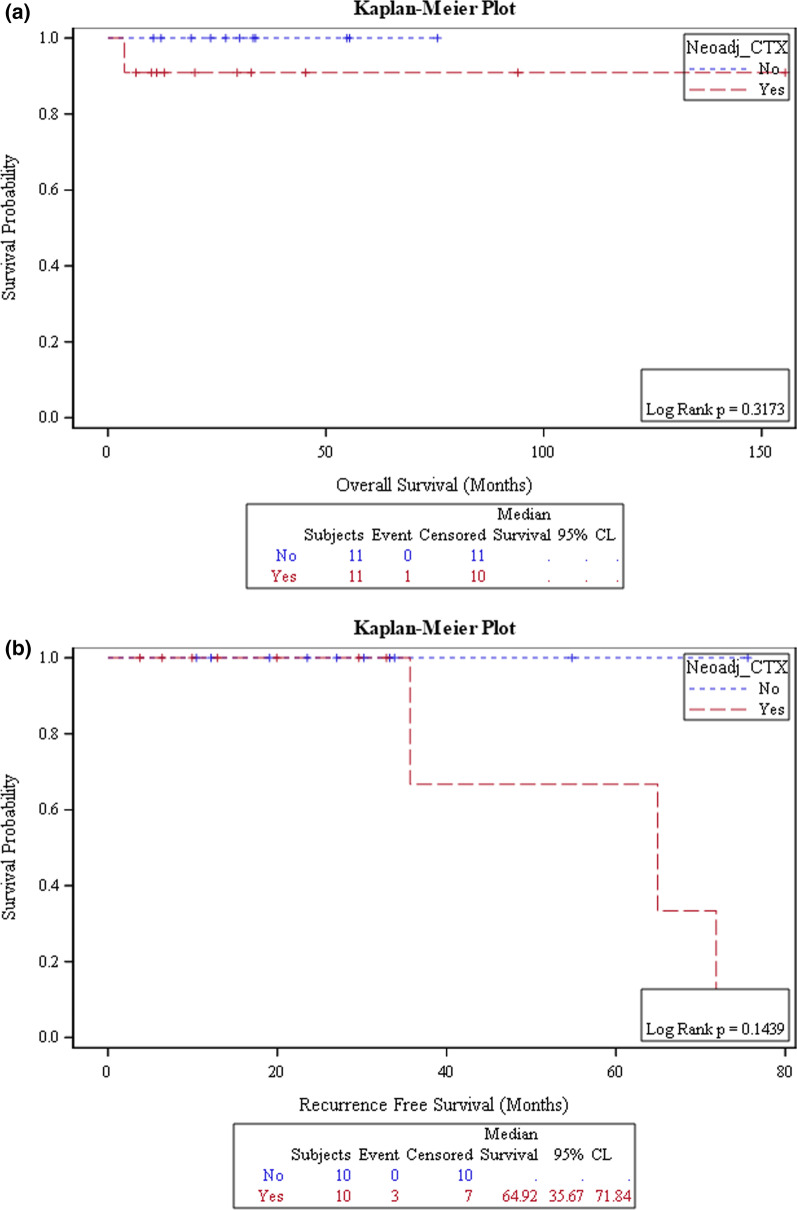
Kaplan Meier curves showing overall survival and recurrence free survival between those who had neoadjuvant therapy and those who did not receive neoadjuvant therapy

There was only a single documented death in the pre-operative chemotherapy group. This patient underwent surgical resection one year after receiving definitive chemoradiation outside our center. Consequently, median survival could not be defined. A time-dependent survival analysis of surgical techniques encompassing overall survival (OS) and recurrence-free survival (RFS) was done. An overall survival rate of 95.65%, yielding a mean survival of 115 months (4–125). Recurrence occurred in 22% (n = 5). The NACT group had a higher risk for recurrence (4; 33%) with a mean survival of 43.8 months in comparison to 59.6 months in those who did not receive induction therapy (Fig. 3).

## Discussion

We are presenting a study in which we retrospectively compared between two groups of thymoma patients. Although our study group number is small but we combined both radiological and pathological tools for better assessment of neoadjuvant chemotherapy effect on tumor burden, and we found that the radiographic and histopathological effect of NACT on thymic tumors is minimal especially on stage III with the greatest variation in tumor burden is in Stage IVa, and did not have any significant oncological outcome effect.

Thymoma, which is the most common anterior mediastinal tumor presents with chest pain, cough and dyspnea and can be accompanied by autoimmune disorders like myasthenia gravis and pure red cell aplasia. One literature described a patient with autoimmune chronic pancreatitis who presented with steatorrhea beside amenorrhea resulting from high gonadotrophin levels [8]. Thymectomy is an optimal choice for treatment but in some cases, the advancement of the tumor and the invasion into adjacent structures open the gate for multimodality treatment of neoadjuvant chemotherapy, surgery, and radiotherapy, in turn, to shrink tumor size and make it possible to resect the tumor with negative margins.

Locally invasive thymic tumors have been found to be responsive to platinum-based therapy with an 80–90% response rate [9], ranging from PAC (cisplatin, doxorubicin, and cyclophosphamide), ADOC (Adriamycin, cisplatin, vincristine, and cyclophosphamide) and CEVP-16 (cisplatin, epidoxorubicin, and etoposide). The response was determined based on two modalities in different studies, radiologic and pathological evaluation.

Radiologic evaluation using RECIST criteria; Berruti et al., reported an (83.3%) PR [8], Lucchi et Al., reported PR in 20 out of 30 patients (66%) and 2 of 30 (6%) CR [10]. On the other hand, Fornasiero reported 30% CR and one PR [11]. Although the mean change in tumor volume in our study was higher than that reported in the literature, it was not statistically significant. Suh et al., using 3D reconstructed imaging, described a significant change in tumor volume of 125 cm^3^ (p = 0.001) [12]. One paper found that radiologic evaluation was not a completely reliable method to judge tumor response [13]. Which has guided other papers to use the pathological evaluation response, indicating necrosis, cystic changes, hemorrhage, and calcification. A limitation of this method rose since the fact that all these histological findings can be found in untreated tumors which will make it difficult to distinguish a chemotherapy effect from untreated tumor findings [14].

Cisplatin-based regimens display minimal effect on achieving negative resection margins [12]. Similarly, although Berruti et al. reported a high response rate, only one of the six patients displayed negative surgical margins [15]. Other papers reported high R0 rates, Mineo et al. reported 51% [13] and Lucchi reported 23 cases of 30 means 76% [10]. Therefore, NACT may prove its utility in preoperative debulking of the tumor in preparation for surgical resection.

Predictor factors in invasive thymoma; combined p27 low, p21 low, and p53 expression proved a predictor of poor response to neoadjuvant chemotherapy p = 0.001 [16]. Histopathologically, Weissferdt Et al. found a higher tumor response rate in tumors that displayed an extensive lymphocytic component, WHO type B1 and B2 [14]. Additionally, pathological stage T3 and smaller tumor size of less than 5 cm have shown better response [17]. In contrast, although not statistically significant we found a higher portion of necrosis in stage IVA (56%) when compared to stage 3 (23%) (p = 0.152).

Reported side effects were nausea and vomiting, alopecia, leukopenia, cardiac toxicity, and infection [10]. Postoperative morbidity was higher in the NACT group vs. the surgery alone group (p < 0.03), expressing respiratory failure, pneumonia, extensive reconstruction (P = 0.05), and complete resection [13]. Mortality due to metastasis, myasthenia complications, myocardial ischemia, and pulmonary fibrosis [10, 13]. Speaking of myasthenia gravis, one literature did not report any deterioration due to chemotherapy and attributed it to effective medical therapy [10].

The literature illustrates no significant difference in overall survival among patients who underwent primary surgery vs neoadjuvant chemotherapy (p = 0.285) [12, 18, 19]. On the other hand, Yamada et al., who studied stage III patients, observed less favorable outcomes in patients given neoadjuvant chemotherapy [20]. Long-term survival was negatively affected by the triple combination of P27, P21, and P53 and incomplete resection (p < 0.0001) [10].

Lastly, we are aware that our definition of pathological response may result in an underestimation of the results. According to the literature, the pathological response does not rely entirely on necrosis as histopathological changes were found to encompass cystic changes, hemorrhage, histiocytic proliferation, and calcifications [21].

Our study has limitations, (1) it was conducted in single tertiary cancer center but King Hussein Cancer Center is considered one of the main cancer centers in the Middle East and the region, receiving multiple referrals from neighboring countries. (2) the study cohort was small and that can be explained by the rarity of the disease. (3) the retrospective design of the study which can only be utilized in such cases due to inability to perform randomization on such population.

## Conclusions

Induction chemotherapy in locally advanced thymoma patients' theoretical role to increase the chance of R0 resection still remains controversial. Despite our small study group number, we found that the radiographic and histopathological effect of NACT on thymic tumors is minimal with the greatest variation in tumor burden is in Stage IVa. However, regardless of stage, NACT was not found to significantly improve oncological outcomes compared to upfront surgery. With all above-mentioned variable results, more studies are required to adjust guidelines for NACT, but due to the rare epidemiology of this tumor it will be difficult to induce a clinical trial but prospective institution experiences.

## Data Availability

The datasets used and/or analyzed during the current study are available from the corresponding author on reasonable request.

## Abbreviations

TETs: Thymic epithelial tumors
NACT: Neoadjuvant chemotherapy
MG: Myasthenia Gravis
CPG: Clinical Practice Guidelines
CT: Computed tomography
RECIST: Response evaluation criteria in solid tumors
CR: Complete response
PR: Partial response
SD: Stable disease
PD: Progressive disease
VATS: Video assisted thoracoscopic surgery
OS: Overall survival
RFS: Recurrence-free survival
